# Conceptualising public mental health: development of a conceptual framework for public mental health

**DOI:** 10.1186/s12889-022-13775-9

**Published:** 2022-07-23

**Authors:** Jennifer Dykxhoorn, Laura Fischer, Becca Bayliss, Carol Brayne, Liam Crosby, Bobbie Galvin, Emma Geijer-Simpson, Oliver Jones, Eileen Kaner, Louise Lafortune, Michael McGrath, Paula Moehring, David Osborn, Mylene Petermann, Olivia Remes, Ami Vadgama, Kate Walters

**Affiliations:** 1 Department of Primary Care and Population Health, UCL Division of Psychiatry, 6th Floor, Wing B, Maple House, 149 Tottenham Court Road, London, W1T 7NF UK; 2 Division of Psychiatry, UCL, London, UK; 3 Cambridge Public Health, University of Cambridge, Cambridge, UK; 4 FUSE, University of Newcastle, Newcastle, UK; 5 The McPin Foundation, London, UK; 6 Camden and Islington NHS Foundation Trust, London, NW1 0PE UK

**Keywords:** Mental health, Public mental health, Indicators, Conceptual framework, Co-production

## Abstract

**Background:**

Numerous determinants have been linked to public mental health; however, they have not been brought together in a comprehensive conceptual framework. The goal of this work was to bring together academic research, practitioner expertise, and public perspectives to create a public mental health conceptual framework.

**Methods:**

The development process proceeded in four stages. First, we identified a comprehensive list of potential determinants through a state-of-the-art academic literature review, grey literature review, and created mind maps created by peer researchers. Next, we conducted in-person workshops, consultations, and an online survey with academics, practitioners, policy makers, and members of the public to review the potential determinants, nominate additional determinants, and prioritise determinants by importance for understanding public mental health. This iterative process resulted in the final list of determinants contained in the framework. We then conducted rapid reviews to define each determinant and to identify key research, interventions, and resources. Finally, we worked with a design team to visualise the conceptual framework as an online tool and printable infographic.

**Results:**

We found substantial overlap between sources reflecting a shared understanding of the key drivers of public mental health. The unique determinants that emerged from each data source highlighted the importance of using multiple sources to create a comprehensive model. 72 potential determinants were prioritised through stakeholder consultations, resulting in a final list of 55 determinants and organised into four levels: individual, family, community, and structural.

**Conclusions:**

This is the most complete conceptual framework for public mental health to date, bringing together academic research, policy and practitioner views, and lived experience perspectives. The co-production processes and tools we used provides a template for researchers looking to include multiple perspectives in their research.

The conceptual framework draws together current knowledge on each determinant, but also highlights areas where further research is needed to better understand the relationship between each factor and mental health, which can inform the research agenda. This online tool and infographic can be used by practitioners to identify interventions for promoting mental health, and by the general public as a resource to increase awareness of the broad factors which shape public mental health.

**Supplementary Information:**

The online version contains supplementary material available at 10.1186/s12889-022-13775-9.

## Background

Public mental health can be defined as the science and art of promoting mental health and well-being and preventing mental health problems through organised efforts of society [1]. The public mental health approach acknowledges that a wide range of determinants across individual, family, community, and structural levels contribute positively or negatively to mental health and well-being [2–4]. The ‘organised efforts of society’ are all public health interventions that aim to improve population mental health by intervening at one or more levels. Interventions might include, for example, individual-level knowledge and skills training for individuals [5], family-level parenting skills programmes [6], community-level efforts to altering aspects of the built or natural environment [7], or societal-level adaption of policies or norms improve the mental health of the population across the life course. Importantly, these determinants are interconnected in complex ways. Effective public health efforts work across levels and simultaneously address determinants at multiple levels.

The British government has recognised the need for a stronger focus on mental health, including wider actions to improve mental wellbeing [8]. The School for Public Health Research (SPHR), a partnership of eight leading centres of academic public health research excellence across England, identified the need for additional research in public mental health. SPHR established the Public Mental Health (PMH) Programme in 2018 in order to generate evidence that will guide public health policy and practice [9].

Despite the growing recognition that mental health is central to public health strategies, there remain important gaps of high-quality evidence to underpin public mental health policies and interventions. Notably, our state-of-the-art review [11] found several theoretical models relevant to public mental health but did not identify an agreed overarching conceptual framework for public mental health, which brings together the wider determinants. We sought to address this gap by developing a conceptual framework for public mental health. The aim of this research was to bring together expertise from academic research, public health practitioners, policy makers, and members of the public to identify the key determinants of public mental health and to create a public mental health conceptual framework. This paper outlines the process of developing a conceptual framework for public mental health to provide a template for future work and policy.

## Methods

The development process proceeded in four stages: (1) evidence/policy reviews and public mind maps to identify a comprehensive list of potential determinants; (2) consultations to prioritise determinants; (3) scoping searches to define determinants and identify resources; and (4) graphic design and illustration to visualise the framework and create online and printable outputs. This research received ethical approval from the UCL Research Ethics Committee and all research involving human data was conducted in accordance with UCL institutional guidelines.

### Stage 1: Reviews and public consultation to identify a comprehensive list of potential determinants

We conducted a ‘state-of-the-art’ academic literature review, a grey literature scoping review, and consultations with members of the public to identify a comprehensive list of potential determinants of public mental health.

We completed a state-of-the-art academic literature review [10] in order to summarise the current state of knowledge on the determinants of public mental health. This review was registered on PROSPERO (CRD42019138753) and covered academic literature published between January 2010 and May 2019 in Medline, Scopus, and PsycINFO [11]. A two-step screening process was used to identify relevant literature, including title and abstract screening, followed by full-text screening. Data were extracted using a standardised form by two authors to identify the determinants and frameworks for public mental health. This state-of-the-art review is in preparation for publication (pre-print version available from authors).

We further conducted a grey literature search using scoping internet searches and consultations with experts to identify key governmental and non-governmental reports, policies, and guidance documents on public mental health and its determinants. We focused on documents from the United Kingdom, but also included key documents from international non-governmental organisations (e.g., the World Health Organisation). These searches were supplemented with forward citation tracking and snowballing techniques to find linked policies and reports. We extracted 27 relevant documents, which were reviewed by academic and peer researchers (JD, MM, OJ, AV, GS). Details of each report and identified determinants were summarised in a standardised data extraction form, to ensure consistent tracking across reviewers (Supplement A). This included a list of determinants identified to date with free-text space to add additional determinants. Reviewers noted which determinants were mentioned in each document and if there was discussion of the factor being modifiable through public health action. New determinants that were not included on the extraction form were added as emerging determinants in the free-text space.

### Co-production with peer researchers

We recruited a team of eight peer researchers who had diverse lived experiences of the determinants of mental health (“experts by experience”) and perspective on how these factors have impacted their mental health. These peer researchers were recruited through social media and outreach. While acknowledging that a complete spectrum of experiences could not be captured in a small team, we reviewed all applications to ensure the peer researchers represented a range of experiences across age, gender, ethnicity, and geographic region.

The members of public selected to join our project as experts by experience were consulted to determine their preferred terminology to capture their role and contribution to the programme. They agreed that the term “peer researchers” best captured both their perspectives which were shaped by their life experiences, and their role as embedded researchers who were actively engaged in the project. The peer researchers were embedded in the research team and contributed to all stages of the project, from developing mind maps to identify potential determinants, co-facilitating public consultations, reviewing academic and grey literature alongside academic researchers, informing the content of the online tool, supporting user testing, and contributing to dissemination activities.

### Public consultation

We conducted a workshop with members of the public to create a collaborative mind map (*n* = 10). Individual and collective maps were drawn without reviewing the determinant lists from the academic and grey literature reviews, so responses would not be limited to factors that have been measured before in research, but rather captured the important determinants from a lived experience perspective. Participants were given information sheets and informed consent forms to review and sign upon arrival at the workshop. Researchers were available to answer any queries.

We reviewed the lists of determinants extracted from the academic review, grey literature search, and public mind maps, removed duplicates, and created a list of potential determinants of public mental health.

### Stage 2: Consultations to prioritise determinants

Next, we conducted in-person and online consultations with a wide range of stakeholders to review, prioritise, and group the potential determinants, resulting in our final list of determinants for inclusion in the conceptual framework.

### In-person workshop

We hosted a workshop for academic, practitioner, policy, and public participants in London in September 2019 (*n* = 38), with representation from our target audiences, including academics, practitioners and policy makers, and members of the public. All participants were asked to review an information sheet and give informed consent upon arrival to the workshop. Participants were mixed, divided into small groups, and asked to review the potential determinants list, identify any missing determinants through a gap analysis, and then rank each determinant according to two factors: (1) important and (2) amenable to change. Participants were guided to consider a determinant as *important* if it provided relevant information considered to be meaningful to understanding public mental health for practitioners, academics, and the public [12, 13]. Participants were also asked to consider the strength of the relationship between each determinant and public mental health. Additionally, participants were invited to discuss whether determinants were *amenable to change*, meaning that the determinants could be changed, modified, or controlled through public health practice or policy to improve public mental health [12, 13]. Participants were asked to consider how easy it would be to change this determinant (e.g. efforts to reduce poverty) or change the relationship of the determinant to mental health (e.g. interventions not focused on changing the determinant itself (e.g. gender identity), but rather focused on reducing stigma and discrimination around gender identity to mitigate the relationship between gender identity and mental health outcomes). When assessing how easy it would be to intervene on each determinant, participants were asked to consider how entrenched the determinant was and if there was evidence for effective interventions that would modify the determinant and its relationship to mental health outcomes based on their knowledge of the research or lived experiences. Participants were asked to identify any missing determinants during a gap analysis, which would be included in the online survey. Finally, participants were asked to discuss what different stakeholder groups would like to see in the final conceptual framework, including focus, format, level of detail, and desired features.

Peer researchers co-facilitated the workshop, including presenting background information, leading discussion, and taking notes and photos during the event, which were summarised in a post-workshop report (available from authors). The research team met to update the determinant list based on the in-person workshop, resulting in a condensed list of determinants organised into four levels: (1) individual, (2) family, (3) community, and (4) structural.

### Online survey

We developed an online survey to further prioritise the determinants and to solicit detailed rankings. The language was reviewed by our peer researchers to ensure accessibility for all participants. Organised by level, we asked participants to rank each determinant on importance to public mental health rated on a 4-point Likert scale from 1 (not very important) to 4 (very important). Participants could suggest missing determinants and make comments in free-text spaces. This survey was circulated widely to our collaborators (*n* = 117), our public mental health stakeholder network (*n* = 215, including academics, practitioners, voluntary sector organisation representatives and members of the public), participants of the in-person workshop (*n* = 38), and on social media accounts (Twitter and Instagram) from SPHR and collaborators. Participants were encouraged to forward the survey to any of their colleagues or peers who may also be interested in providing feedback. Participants who accessed the survey were given participant information and an informed consent form prior to completing the survey, in accordance with our ethics approval.

The survey was open for responses for over a month and106 people participated in the survey. Of those who participated, 93 gave details of their professional role, including 39 academics (41.9%), 30 members of the public (32.3%), 13 from the third sector (14.0%), 11 public health practitioners and policy makers (11.8%), and the remaining 9 (9.7%) selected ‘other’, including clinicians and students. Of the 94 participants who responded to their region of residence, 86.2% (*n* = 81) were from England, 10.6% (*n* = 10) were from Scotland, Northern Ireland, or Wales, and 3.2% (*n* = 3) were from outside the UK. There were participants from each of the nine regions in England, with the highest number of participants from London (30.9%, *n* = 29) followed by Yorkshire and the Humber (12.8%, *n* = 12).

The research team summarised the results, including calculating a mean importance score for each determinant and collating free-text comments and suggestions. The research team reviewed the rankings and suggested determinants, and discussed revisions to ensure coherence, consistency, and readability, resulting in the final determinants list. Determinants were further divided into groups within each of the levels to make the list more manageable.

### Stage 3: Rapid scoping searches to define determinants and identify resources

Using the final determinants list, we conducted rapid scoping searches to generate detailed information for each determinant. Specifically, we (1) generated definitions, (2) explored the relationships between each determinant and mental health outcomes, (3) identified existing interventions that aim to modify the determinant or its relationship with mental health, (4) explored lived experience accounts of the determinant, and (5) identified intersectional links to other determinants in the framework.

### Generating definitions

We limited each definition to 75 words and made every effort to ensure these were written in plain language, so they would be accessible to a wide audience.

### Exploring relationships with mental health

We explored the relationship between each determinant and mental health using scoping searches of academic and grey literature, and coding each as a risk factor, a protective factor, or both (depending on absence/presence and quality).

### Identifying interventions, resources and lived experiences

Whenever possible, we prioritised links to open-access resources over those behind publication paywalls. We used scoping internet searches and conversations with peer researchers, academic researchers, and public health practitioners to identify interventions and resources that aim to modify the determinant (e.g. increase social support) or improve the mental health of those who experience the determinant (e.g. interventions which support carers). We used the same approach to identify lived experience perspectives for each of the determinants.

### Nominating intersectional links

Recognising that the determinants are interdependent and that changes in one area may affect many other determinants, we identified key related factors (*Connected Determinants)*. Two peer researchers and two academic researchers identified connected determinants, which were discussed by the broader research team to identify the top links.

### Stage 4: Design to visualise the framework and create online and printable outputs

We worked with a design team (LF, BG, MP) to develop designs and illustrations to visualise the conceptual framework and create a comprehensive, accessible online tool and a printable infographic summary. The goal of the tool was to provide an overview of determinants of public mental health, with the ability for individuals to “zoom” into each level, group, and determinant to get more details. This approach was informed by the consultations from Stage 1, where our stakeholders discussed the merits of having an overarching picture of public mental health as well as the need for a detailed view which could highlight evidence-based literature and interventions.

We conducted three user testing sessions with three participants in each session. Participants were recruited using social media posts and networks. Expressions of interest were reviewed by members of the project team (JD and BB) to ensure diversity by geographic region, gender, age, and professional background. The invited participants had not been involved in previous consultations, so would be naïve to the project at the start of the session. Participants were asked to review an information sheet and complete an informed consent form prior to attending the session. Participants were instructed to complete several tasks within the online tool (e.g., navigate to a specific determinant or find which determinants are part of the community level). Feedback from these sessions was incorporated into the tool to enhance usability. Next, we held a workshop as part of the SPHR Annual Scientific Meeting, where we presented an early version of the online tool. The 61 participants in the workshop were able to explore the tool and provide feedback on the layout, content, and functionality, which also informed the final product.

## Results

### Stage 1: Reviews to identify a comprehensive list of potential determinants

#### Academic literature review

**Fig. 1:**
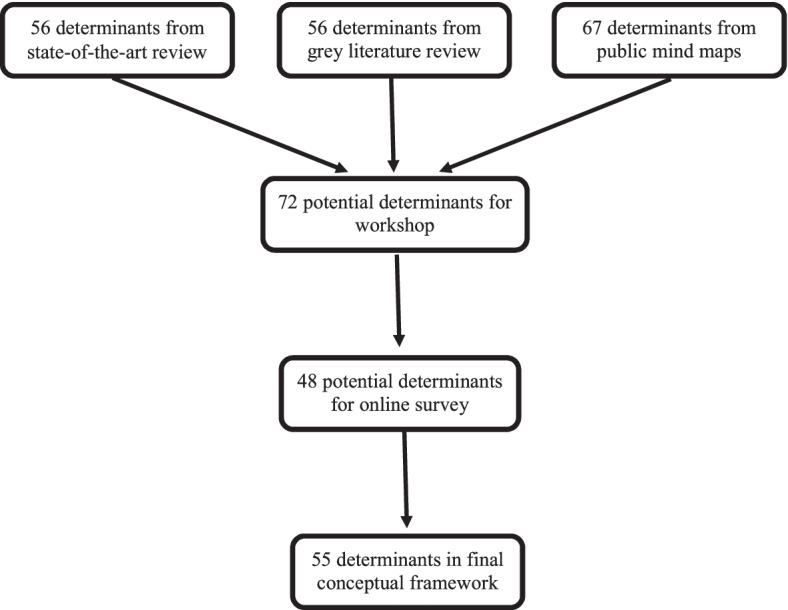
Determinants identified at each stage

Of the 5,170 papers identified in the search, 95 papers met the inclusion criteria and were thematically analysed (protocol published [11], paper in preparation and pre-print available from authors). 56 determinants were identified in the academic review and grouped into four levels: individual, unit, community, and society (Table S1; Fig. 1). The review additionally found that the Socioecological Model and resilience frameworks, like the Resilience Activation Model, were the most common frameworks for describing public mental health in the published literature.

#### Grey literature review

We extracted 27 full-text reports, as identified by scoping internet searches, expert consultation, and snowball citation tracking (Supplement A for full reference list of extracted reports and Figure S1 for excerpt from data extraction form). 73.9% of the reports were from the UK and 26.1% were from international organisations, including the World Health Organisation. Most of the reports focused on the general population (82.6%), with some focusing on population subgroups, including children, young people, adults, and older adults. The grey literature search identified 56 determinants. 43 were similar to those identified in the academic review, and 13 were unique determinants including family structure, migration, nature and ecotherapy, exam stress in students, artificial intelligence, and life transitions (Table S1; Fig. 1).

#### Public mind maps

Mind maps were created both by peer researchers (*n* = 8) and by public participants at an in-person workshop (*n* = 10). The mind maps identified 67 determinants, including many which were similar to those in the academic and grey literature searches as well as several additional determinants (Fig. 1). Notably, the public mind maps identified a range of personal traits and attributes that were not captured in the academic and grey literature reviews, including self-esteem, self-compassion, compassion, kindness, feeling valued, sense of purpose, fulfilment, contribution, autonomy, ability to self-regulate, feeling of choice and control, and ability to experience a full spectrum of emotions (Table S1).

#### Potential determinant list for in-person workshop

The research team reviewed the lists generated by these three sources and identified areas of overlap and divergence. There was substantial overlap between these sources, with many determinants appearing in all three, including income, employment, housing, education, trauma and adversity, resilience, social networks, social inclusion, built environment, neighbourhood deprivation, policies and laws, political structures, discrimination and stigma, media and social media, and social expectations and norms.

There were also several areas where the three sources diverged. As noted above, the public mind maps identified several determinants that had not appeared in the other two sources. The sources also differed in terms of the nuanced detail included in each determinant. For example, employment was identified in all three sources as an important determinant of public mental health, but it was described differently in each. In the academic review, job security, unemployment, and underemployment were identified. The grey literature search included unemployment and underemployment, but also added occupational position and work environment. The public mind maps identified having a job (employment/unemployment), fair conditions, job suitability, job satisfaction, work-life balance, and work environment. Similarly, while the grey literature review identified education and school environment as determinants of public mental health, the public mind maps explored aspects of education, including accessibility, inclusion, quality, and completion, in addition to the educational environment. The mind maps also drew out the importance of access to amenities, health promoting activities, and health and social care. This emphasis on the availability and accessibility of services was missing from the grey literature and only partially captured in the academic search.

There were several specific determinants identified in the academic review that were much broader in the other sources. For example, the academic search found several specific determinants that related to trauma and adversity, including early life sexual, emotional, and physical abuse, intimate partner violence, economic abuse, lifetime trauma, history of rape or stalking, and physical assault. These were broadly identified as trauma and adversity in the grey literature search and divided according to the life course and distinct impacts on mental health (childhood adverse experiences and adulthood adversity and trauma) in the public mind maps.

One determinant, commercial determinants of health, including the impact of decisions made by private companies on mental health, was identified in both the academic and grey literature search but did not appear in the public mind maps.

The determinants from all three sources were combined into a list of 72 potential determinants, which was used for the in-person workshop in Stage 2 (Table S1).

### Stage 2: Consultations to prioritise determinants

#### In-person workshop

In small groups of 12–15 participants, participants reviewed the list of potential determinants and discussed how these might be prioritised by importance and amenability to change. These discussions varied by group, although all groups acknowledged that it was difficult to prioritise the long list of potential determinants.

One small group, composed of academics and members of the public, identified the following 13 determinants as the most important and amenable to change: early years, social relationships, social networks, loneliness, individual aspirations, employment, housing, income, education, resilience, stigma and discrimination, support services, and at-risk groups.

A small group of practitioners and policy makers indicated that importance and amenability to change should be assessed separately at the national and local levels. This group identified the following determinants as both important and amenable to change at the national level: education, housing, aid, gender/sex, media/advertising, employment, discrimination/stigma, social fragmentation, and physical health. At the local level, they rated loneliness, bullying, health behaviours, access to employment/job conditions, social support/social networks as important and amenable to change. All groups agreed that genetics and biological factors were the least important and the most difficult to change through public health action.

During the gap analysis, 12 additional determinants were added to the list, including language and fluency, alcohol and substance use, educational transitions, general practitioners’ knowledge of mental health (Table S1). These were included in the subsequent list for online consultation.

The small groups also discussed what format and level of detail would be most useful to include in the conceptual framework, comparing strengths and limitations of frameworks from other areas. In order to balance the desire for simplicity and detail, it was suggested that the conceptual framework should have an overarching summary view which highlights the breadth of determinants influencing public mental health, and a more detailed view which would include evidence, interventions, and resources.

#### Online survey

Based on all responses, we calculated mean importance scores between 1 and 4. Overall, most determinants scored above 3, indicating that, on average, most determinants were considered to be moderately to very important. The determinants receiving the highest importance scores at the individual level were trauma (3.74, 95% CI 3.61–3.87), housing (3.69, 95% CI 3.59–3.79), income (3.64, 95% CI 3.59–3.79), and employment (3.61, 95% CI 3.50–3.72). At the family level, early life attachment & parenting received the highest importance score (3.62, 95% CI 3.50–3,74). The determinants rated as the most important at the community level were social support & networks (3.66, 95% CI 3.54–3.79), access to health & social care (3.59, 95% 3.46–3.72), and social inclusion & cohesion (3.52, 3.38–3.66). At the structural level, displacement (3.62, 95% CI 3.47–3.77), inequality & inequity (3.56, 95% CI 3.40–3.72), and welfare system (3.52, 95% CI 3.20–3.54) received the highest importance scores.

Several determinants received scores below 3, indicating that, on average, participants viewed these as less important. The determinants receiving the lowest mean importance scores were religion, spirituality & faith (2.18, 95% CI 2.03–2.33), hobbies (2.26, 95% CI 2.49–2.81), and genetics (2.51, 95% CI 2.33–2.69). No determinant scored below 2, indicating that there were no determinants that were viewed as unimportant to our understanding of public mental health. Further, the scores were highly clustered, indicating that there was broad agreement that the identified determinants were relevant to public mental health.

Participants were invited to nominate any determinants that were missing from the list which they considered to be very important for public mental health. These were added in free-text fields and summarised by the research team.

The research team, including academic and peer researchers, reviewed the rankings and additional determinants, discussed editorial changes which would make the determinant list more readable, and created sensible groupings of determinants to make the list more manageable. This process resulted in the final list of 55 determinants, organised into 15 groups, and embedded within four levels (individual, family, community, and structural) (Fig. 1; Table 1).

**Table 1:** Determinants in the public mental health conceptual framework

Individual	Family	Community	Structural
*Trauma & adversity*	*Family dynamics*	*Systems & services*	*Broad factors*
Adverse childhood experiences	Attachment	Health & social care	(In)equality & (in)equity
Adulthood trauma	Parenting	Public & community services	Climate change
Bullying	Family connectivity	Criminal justice system	Displacement
*Physical & psychological health*	Extended family relationships	*Social environment*	*Industry*
Genetics & biological factors	Discord & conflict	Social support & networks	Commercial factors
Prenatal & perinatal factors	*Family structure*	Social inclusion & cohesion	Media & advertising
Physical health	Caring responsibilities	Civic engagement	*Government & political*
Health behaviours	Intergenerational (dis)advantage	Mental health awareness	Economic conditions
*Life experiences & opportunities*	Household composition	*Geographic & physical environment*	Government policies & legislation
Life transitions	Marriage, civil & domestic partnerships	North–South divide	The welfare system
Migration		Built & natural environment	Political structures & climate
Hobbies & leisure time		Urban/ rural/ remote differences	Global politics & events
*Identity*		Neighbourhood deprivation	*Norms & rights*
Ethnicity & culture		Community safety	Discrimination & stigma
Gender, sex, gender identity & sexual orientation			Social & cultural norms
Religion, spirituality & faith			Human rights & social justice
*Personal traits*			
Resilience			
Sense of self			
Personal aspirations & ambitions			
Individual autonomy			
*Sociodemographic*			
Income			
Housing			
Education			
Employment			

#### Stage 3: Scoping searches to define determinants and identify resources

Using rapid scoping searches, we developed definitions for each determinant in the final list and explored their relationships with mental health. These definitions were reviewed by academic and peer researchers to ensure the definitions fully captured the meaning of each determinant and were understandable to the public. Each determinant was represented on a card which included a succinct definition, an indication of whether it was a risk factor for mental health, a protective factor, or both, a summary of the research linking the determinant to mental health outcomes. In total, 17 determinants were coded as risk factors, 7 as protective factors, and 31 as both risk and protective factors. We aggregated key literature, interventions, resources, and lived experiences with the goal of providing a source for practitioners, academics, and the public who are looking for evidence and resources related to public mental health. Where possible, we identified up to three interventions, resources, and lived experience perspectives for each determinant. These were identified in consultation with peer researchers and academics, and scoping internet searches. When more than three were identified, we prioritised resources that were openly available (open access) and those which reflected the UK context.

The interconnected nature of the determinants was identified in our initial consultations and finding a way of representing this intersectionality was an important aim of our framework. We created a ‘connected determinant’ section on each determinant card which provides short-cut links to factors that are directly related to the determinant in question. Two researchers with lived experience (LF, MP) and two academic researchers (JD, PM) independently identified connected determinants that were closely related to each determinant. These lists were discussed by the research team, resulting in up to 9 connected determinants on each determinant card, represented as suggested short-cut links to other areas of the framework.

#### Stage 4: Design and illustration to create interactive online tool and printable infographic

As described in Stage 2, the in-person workshop included discussion of what the final conceptual framework should include to be useful and meaningful to our stakeholders. During this discussion, we reviewed frameworks from other fields, highlighting the strengths and limitations of approaches from simplified schematics to complex systems maps. The overall recommendation was to create an online tool which includes a broad view that showcases the wide range of determinants impacting public mental health and the ability to zoom in to get more detailed information on each determinant.

**Fig. 2:**
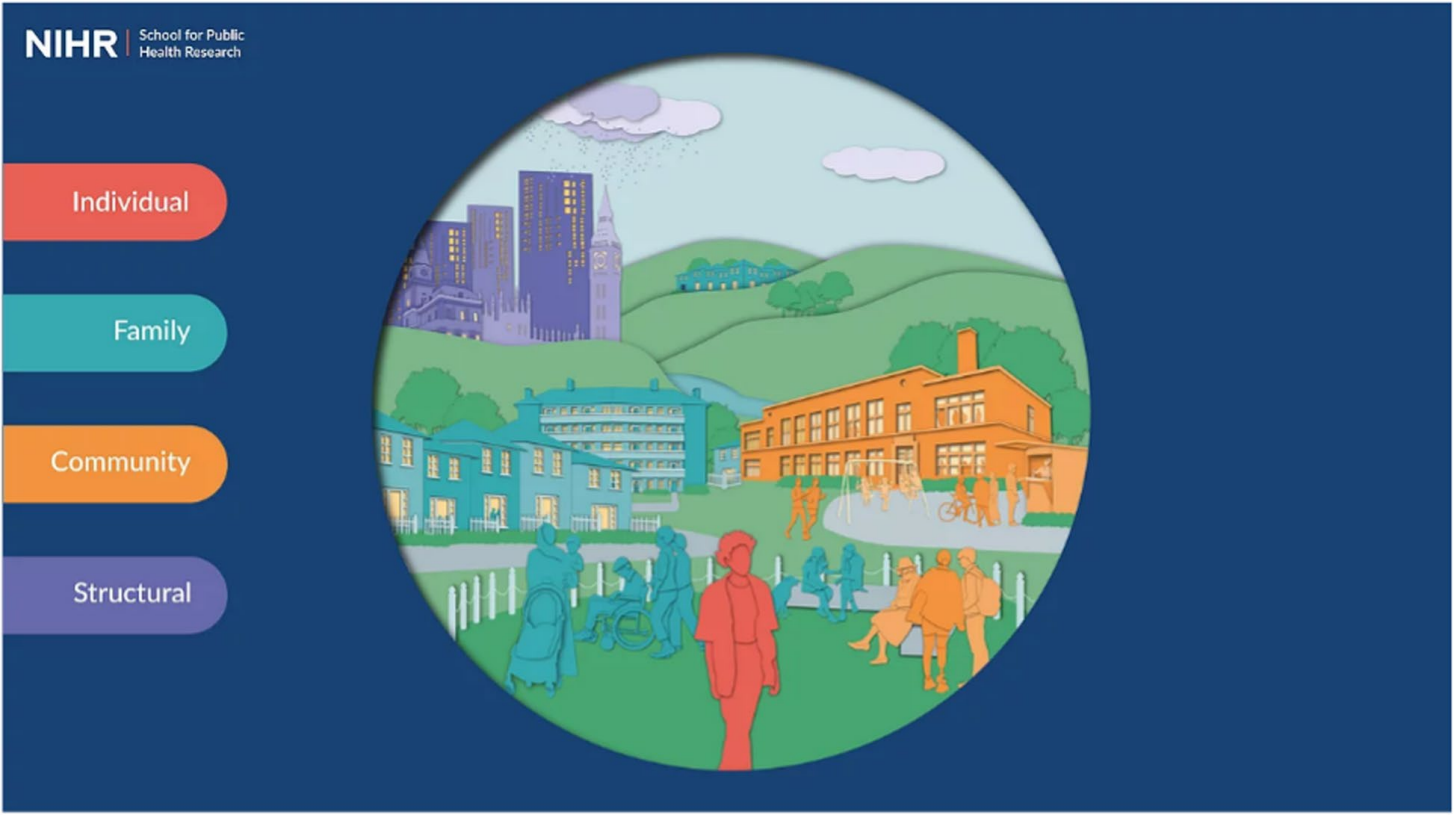
Conceptual framework home page

**Fig. 3:**
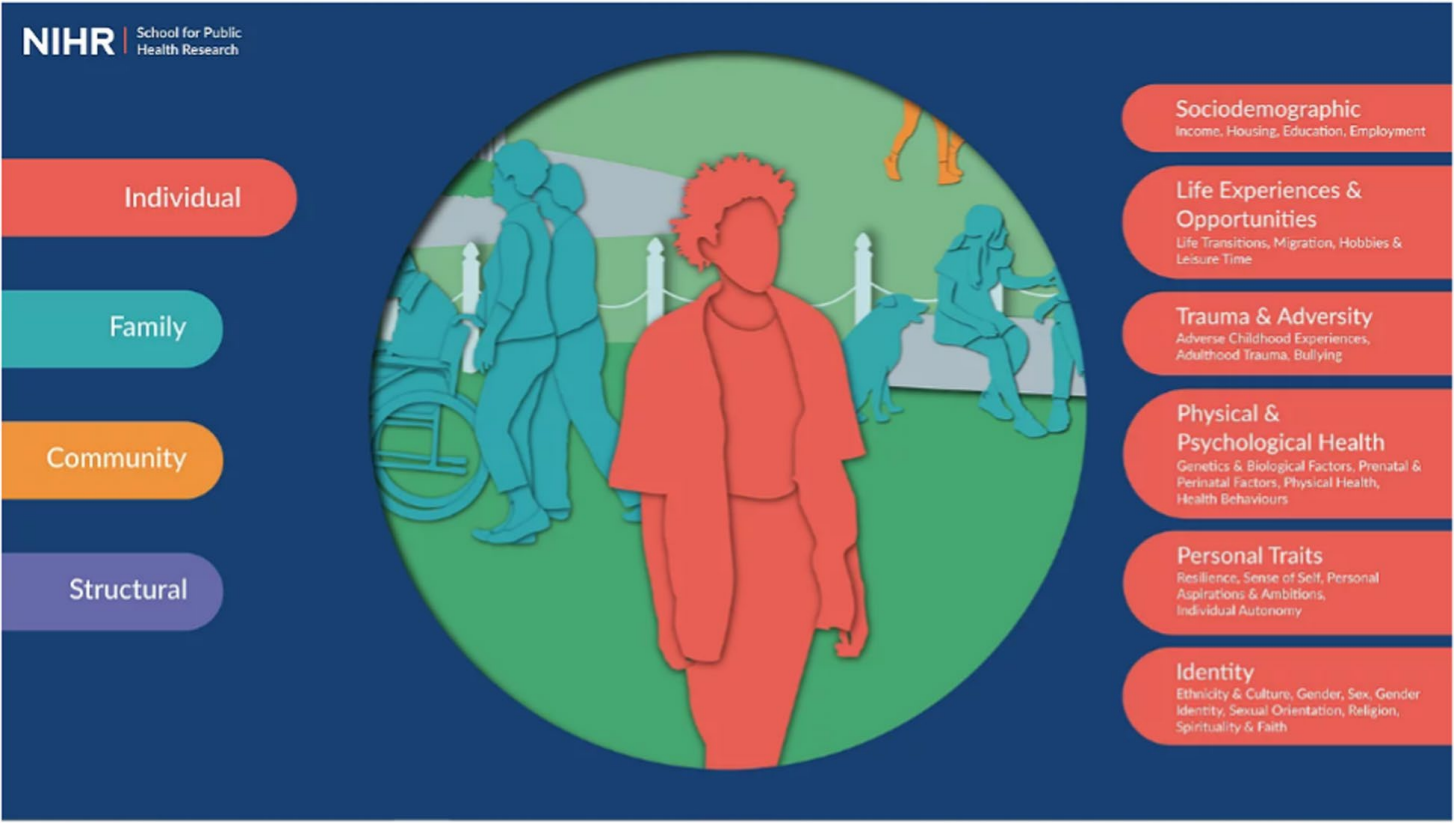
Example of level – individual

**Fig. 4:**
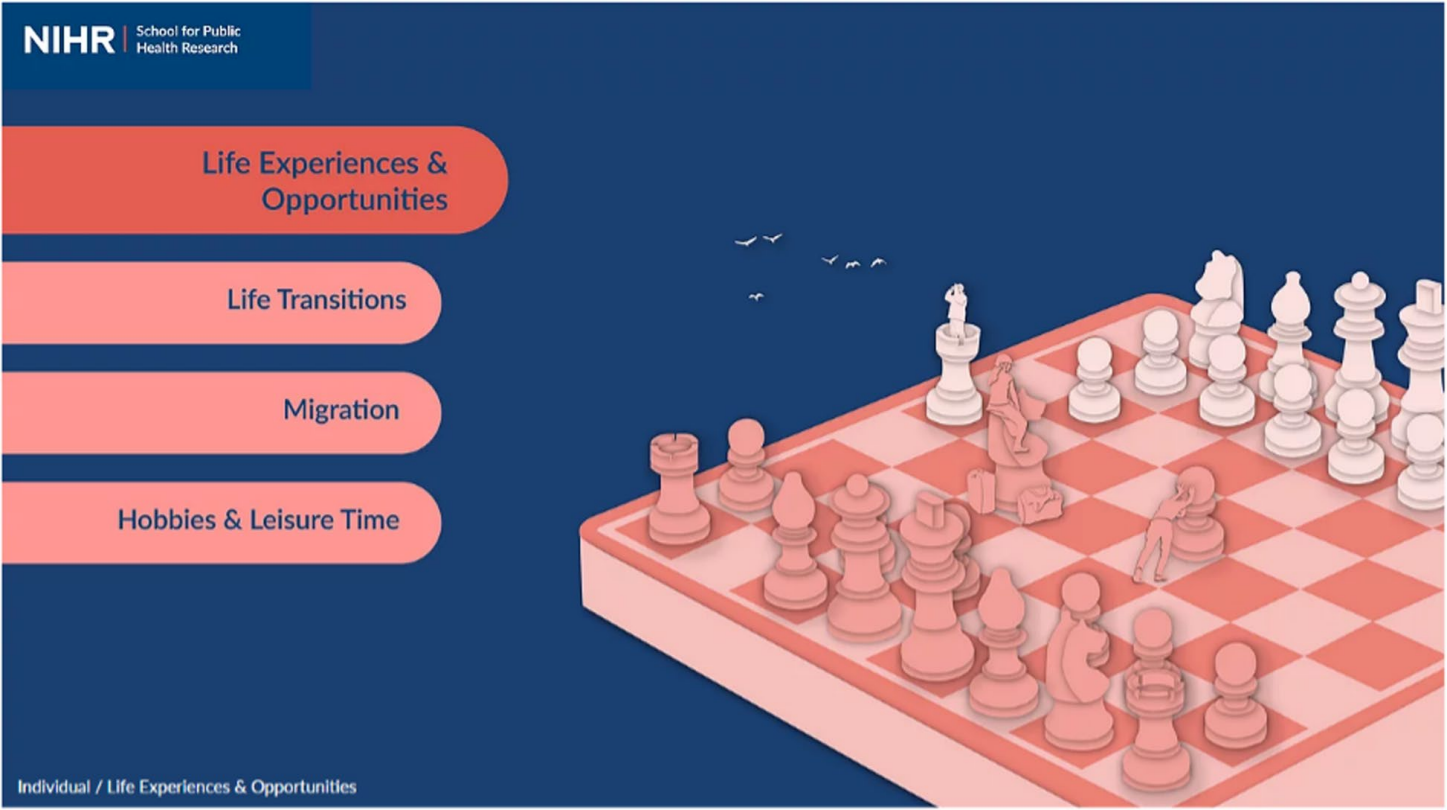
Example of group—life experiences and opportunities

**Fig. 5:**
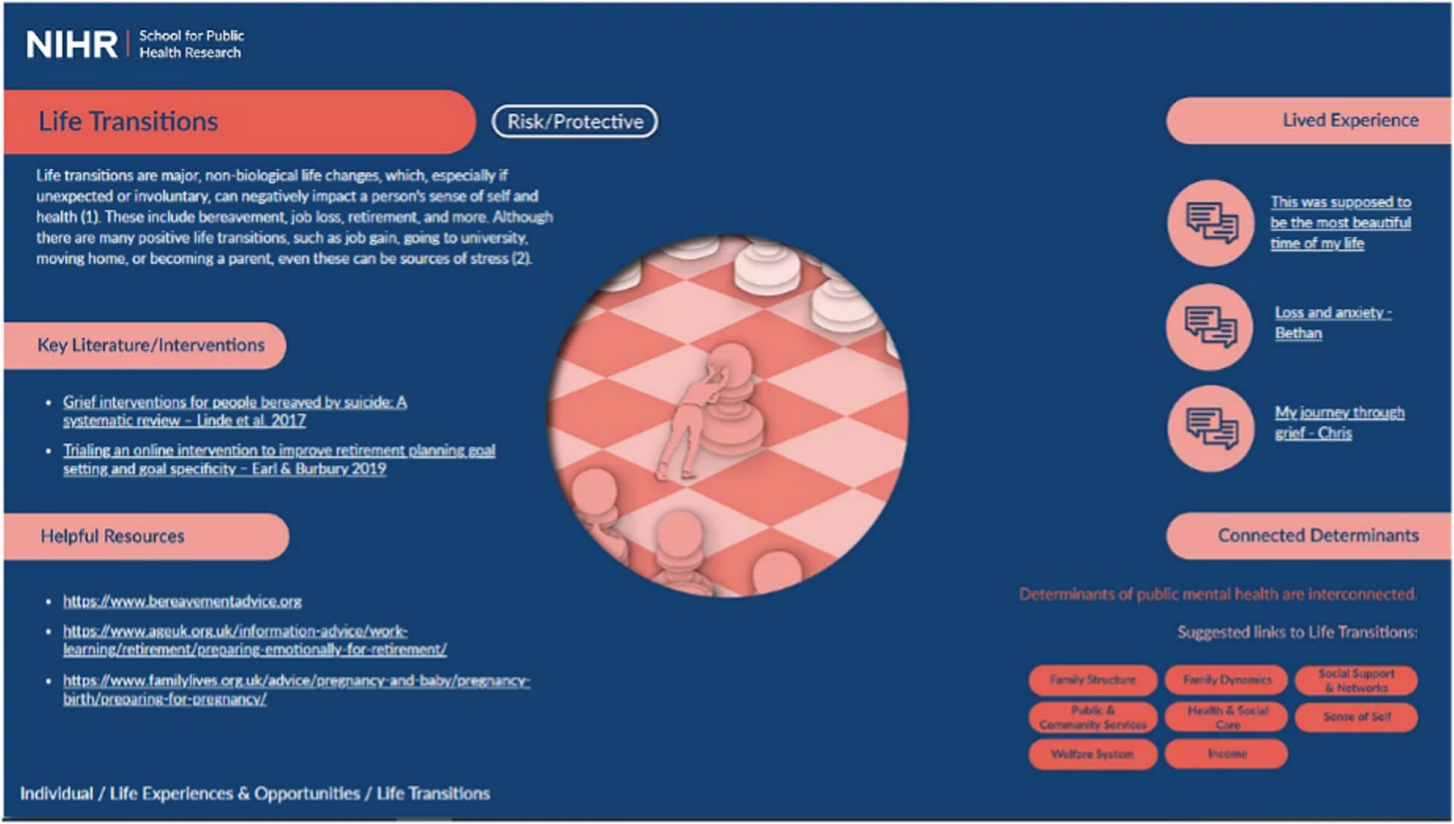
Example of determinant– life transitions

To achieve this vision, we partnered with a design team to develop an online tool hosted on a website (www.publicmentalhealth.co.uk). The site includes a home page which depicts the four levels of the framework: individual, family, community, and structural (Fig. 2). From this page, you can zoom in to each level to see additional details. Figure 3 shows the individual level of the framework, including grouped determinants. From this level, you can zoom in further to see each group (Fig. 4) and finally each determinant (Fig. 5).

The design team worked closely with the research team to create a layout which worked to showcase all the details for the framework, using colours and illustrations to depict and differentiate levels, groups, and determinants.

Once the tool was developed, we invited stakeholders to participate in user testing sessions. We held three semi-structured user testing sessions. These 1.5-h sessions were attended by 9 participants and facilitated by members of the research and design teams (LF, BG, JD, PM).

The overall impression of the tool was positive in these sessions, with enthusiasm for the tool and an indication that the layout is visually engaging and includes useful information. Participants were asked to navigate the tool and to find specific indicators. Participants gave suggestions to improve accessibility and ease of navigation by increasing the font size, adding text to navigational buttons, adding an index, and making small modifications to the graphics.

We presented the draft tool during a workshop at SPHR’s Annual Scientific meeting in April 2021. 61 people participated in an online workshop. After an introduction to the online tool, participants were invited to explore the tool and make suggestions on how to make the final version more useful and accessible.

The research team and design team reviewed suggestions from the user testing sessions and workshop and adapted the tool to respond to these comments. We also created a printable summary of the webtool (Appendix B). Following these updates, we have finalised the framework, which was formally launched in October 2021.

## Discussion

The public mental health conceptual framework brings together academic literature, grey literature, and consultations with practitioners, policy makers, and members of the public. This paper provides a detailed description of the development process of a public health tool grounded in academic research, lived experiences, and practitioner perspectives in order to create a comprehensive view of public mental health, produced in an accessible format. The iterative consultations helped us shape the framework to ensure it is informative and meaningful to our stakeholders.

### Identifying determinants using multiple sources

This comprehensive conceptual framework was made possible by bringing together information from multiple sources. One of the interesting aspects of this research was to observe what determinants were identified across all three data source and which ones were unique to a particular perspective. While there were many determinants that appeared in all sources, the public mind maps identified many determinants which had not been discussed in the academic or grey literature. This additional richness achieved through extensive public consultation speaks to value that meaningful public involvement can have in the development of conceptual frameworks, which has not been described in the development of other frameworks. Further, this project has demonstrated that mind maps may be an accessible and useful tool for soliciting public input into future research projects.

### Defining determinants

This is the first time many of these determinants have been defined in relation to mental health, representing an important contribution to our understanding of the drivers of public mental health. A further challenge was to determine the appropriate language for the tool, which will communicate the scientific and technical aspects into accessible language that meets the needs of our stakeholders.

### Prioritising determinants

During our stakeholder workshop, we asked stakeholders review the potential determinants list, identify any missing determinants, and prioritise potential determinants based on their importance to public mental health and their amenability to change through public health action. When sorting determinants by importance to public mental health, many stakeholders asked if we had estimates of the strength of the relationship between each potential determinant and mental health outcomes. A limitation of our approach was that we did not have systematic estimates of these effects, which meant participants had to rely on their pre-existing knowledge of the literature or personal experiences to provide their view on the importance. Ranking the determinants based on amenability to change through public health action was similarly difficult. First, public health interventions often lack thorough, long-term evaluation, so the evidence for effectiveness of interventions was limited. Further, many factors may be amenable to change, but constraints around resources cannot be ignored, as resource allocation and political will are critical factors to consider.

Overall, this was a challenging task, as participants were asked to comment on 72 potential determinants within a single session. Other prioritisation exercises the authors have participated in have divided potential determinant lists into subsections to be reviewed separately by small groups. While this has an advantage of allowing for more conversation for each potential determinant, it does not provide each participant with an overall view of what is being considered for inclusion in the framework. One of the goals of this exercise was to identify completeness and gaps, which would not have been possible without reviewing the full potential determinant list. Further, there was rich discussion about which level several of the determinants should be included at, which was informative for our later decisions.

The online prioritisation exercise also had challenges. While each participant was able to take as much time as they needed to complete the prioritisation, there was little context provided to inform their decision on how to rank each determinant. The online survey was helpful in that it permitted a higher number of stakeholders to participate, however, there was limited ability to discuss broader conceptual issues or provide context and clarification to participants in the online format.

Overall, asking stakeholders to prioritise an extensive list of determinants during an in-person or online consultation risked simplifying a complex process. Each determinant is intricately linked with other determinants in the framework, so using a prioritisation exercise to justify investing in some areas while dismissing others may ignore the complex system within which determinants arise. In order to address this challenge, we have attempted to embed systems thinking to the way we visualised the connections between determinants and worked to ensure the conceptual framework was able to illustrate the intersectionality of determinants for public mental health.

### Sorting determinants into levels and groups

At the various development stages, we had between 48 and 72 determinants, which became difficult to review and discuss in detail. As mentioned above, within each of the determinants could be multiple additional aspects to consider. To simplify the overall view, we sorted determinants into four levels: individual, family, community, and structural. These four levels had face validity and were similar to existing frameworks for mental health, well-being, and the social determinants [12]. We further divided determinants within each level into 15 groups, which allowed us to create small groups of closely related determinants. However, the process of sorting and assigning determinants to specific levels and groups had some limitations. In particular, there were several determinants which reasonably fit in multiple levels. Each determinant is undeniably linked to factors across the framework and several could be comfortably placed in several levels. We chose to categorise each determinant at a single level for simplicity and accessibility of the tool. We used the connected determinants link to communicate that each determinant is linked to other determinants within the tool.

We also grouped similar determinants within each level. For example, we created a ‘sociodemographic’ group which includes education, employment, housing, and income, which were thought to be closely related to each other. When defining the final list of determinants, we combined several of the determinants in an effort to balance nuanced specificity and the need for simplicity and accessibility. Following the online consultation, the research team met to discuss if any individual determinants could be reasonably combined with another without losing its meaning. Some examples of combined determinants include social support and networks, genetic and biological factors (e.g. age, hormones, brain chemistry), and health behaviours (including physical activity, nutrition, sleep, and substance use). The results are therefore a parsimonious list of determinants which still capture the breadth of factors that affect public mental health.

When observed as a whole, it was apparent that the distribution of determinants was not equal between the levels. While the individual level boasted 21 determinants, the family, community, and structural levels have far fewer: 9, 12, and 13 respectively. While the literature on public mental health widely recognises the importance of higher-level determinants, the preponderance of determinants at the individual level may reflect a bias in measurement and research. Much public mental health research is based on population surveys, which have provided rich information on many individual factors, like socioeconomic status, life experiences, and health behaviours. Few large-scale population studies have included scales which measure social norms, system performance, or political factors. This impacts the amount of evidence we have on the relationship between each determinants and mental health. The paucity of evidence, particularly at the structural level, may be due to gaps in measurement, rather than lack of an important association. Rather than continuing to replicate known associations at the individual level, public health researchers should consider which higher-level constructs may be most relevant to public mental health and how these could be measured. There may be opportunities to develop new measurement approaches or work in multidisciplinary teams to access alternate data sources.

### Strengths

#### Multiple sources of knowledge informed the framework

A key strength of the development process was that we used an inclusive, collaborative process which brought together voices beyond the academic literature to create a co-produced and comprehensive picture of public mental health. Frameworks based solely on academic literature can miss determinants that are important to the lived experience of public mental health, particularly if these determinants are difficult to measure. We brought together well-established determinants from the academic literature and grey literature with expertise from lived experience and public health practice.

The substantial overlap between the determinants identified in academic research, reflected in policy documents, and supported by members of the public demonstrate a wide acceptance of several key determinants of public mental health. This was further shown in the importance rankings from the online survey, where most determinants received an average score of moderately important, reflecting broad consensus of the determinants of public mental health.

The unique determinants identified in each of the sources highlighted the utility of bringing together multiple sources to capture a comprehensive view of public mental health, as a framework based on only one form of evidence would miss important aspects. Notably, the emergence of personal traits and attributes within the public mind maps, including sense of self, aspirations, self-regulation, and sense of contribution were not discussed in the academic or grey literatures. This suggests that there was an evidence gap for the relationship between of personal traits and attributes on mental health outcomes which may require further investigation.

The nuanced detail that was included in the public mind maps was also distinct from the academic and grey literatures in many cases. For example, the mind maps emphasised that myriad aspects of education, including accessibility, inclusion, quality, and completion, were all determinants of public mental health. The predominant measure of education explored in the academic and grey literature was level of education, which might reflect the measure that is most commonly included in surveys and studies but that does not capture the complexity of the relationship between education and mental health.

In the area of trauma and adversity, the academic search had identified literature related to specific types of abuse and trauma, including economic abuse, sexual abuse, emotional abuse, physical abuse, intimate partner violence, rape, stalking, and more. These were captured more broadly by life stage in the other two sources – adversity experienced in childhood and adult trauma and adversity. The level of detail included in the measures from the academic literature may reflect research exploring hypotheses estimating the specificity of trauma type on mental health outcomes, while the focus on overall life course exposures may reflect a broader perspective on how adverse experiences during childhood and adulthood may have different effects on mental health.

The lack of commercial determinants represented in the public mind maps might indicate a relatively low level of public knowledge of how corporations and private businesses can impact mental health. This might represent an opportunity for further public discourse about the role that private corporations have on mental health outcomes and motivate further research to better understand the mechanism linking commercial determinants to mental health.

#### Iterative consultations

The highly consultative process we followed allowed us to ground the framework in research as well as lived experience, public health practice, and policy. This enabled us to capture a broad range of perspectives, which has not previously been done in public mental health. The iterative approach enabled us to revise the determinant list at each stage and encourage conversations across the stakeholder groups to further adapt the framework.

#### Format

The public mental health conceptual framework (www.publicmentalhealth.co.uk) has been created to be a highly visual representation of the drivers of public mental health. These visuals, in combination with the evidence-based research, resources, and lived experience perspectives, has created an innovative and engaging tool. We have developed the framework as an interactive online tool, which includes a simple overview as well as detail within each determinant. These different views allowed us to create a tool which may be useful to a variety of audiences. The extensive user testing and consultation allowed us to adapt the online tool to meet the needs of our stakeholders. We have also created a printable version, which captures the full conceptual framework at this time and is a snapshot of our current knowledge of the determinants of public mental health (Appendix B).

#### Limitations

While we made an effort to create a comprehensive framework which summarised the current knowledge of the determinants of public mental health, there are some limitations to note.

#### Strength of association between determinants and mental health

We used literature reviews and consultations to identify determinants for this framework, but we did not have the capacity to explore the strength of associations between each determinant and mental health. Thus, our framework does not provide information on which determinant was most strongly related to mental health, which might be useful information to inform the likely impact of interventions to modify the drivers of mental health problems.

#### Difficulties representing intersectionality in a meaningful way

From the beginning of this research, we identified that it was critical to represent the intersectionality of determinants in the framework. In the final framework, we included links to connected determinants, but this does not fully capture the interplay between multiple factors across all levels. While the inclusion of the connected determinant links highlights our desire to consider intersectionality, this solution falls short of what could be achieved in a dynamic system map which identifies complex and changing relationships between multiple factors.

#### Limitations around effective interventions

We ran scoping searches to identify key resources and interventions designed to address each determinant. However, the list of included resources is not comprehensive and further exploration of the evidence around effective interventions would strengthen this tool.

#### Investment in development and sustainability

This tool has represented a significant investment of time and resources. We initiated this project in Spring 2019, and it has taken more than two years to complete the research, consultations, and design. This has been a resource-intensive project, requiring skills from numerous collaborators. Early feedback from our stakeholders has indicated that they find the tool interesting, engaging, and potentially useful for their work by highlighting resources, interventions, and lived experience perspectives. However, this iterative consultative process and the interactive visual product may not be suitable for future research projects which have shorter timelines and fewer resources.

#### Sustainability

This conceptual framework is more complete than others that are currently available, representing a major step forward in bringing the disparate evidence on public mental health together. However, continued investment would be needed to update the tool as new evidence emerges.

## Conclusions

The public mental health conceptual framework brings together academic research, policy and practitioner views, and lived experience perspectives into a comprehensive summary of public mental health. The 55 determinants were organised into four levels (individual, family, community, structural) had been identified through iterative consultations with our stakeholder groups. This framework was co-produced with public and practitioner stakeholders, whose contribution has allowed us to create the most comprehensive framework for public mental health to date, reflecting the value of meaningful public and practitioner involvement in research.

As the most complete conceptual model of public mental health, this framework highlights overlooked determinants and evidence gaps, which could inform the public health research agenda in the coming years. This framework provides a helpful starting place for public health practitioners working to promote mental health and prevent mental illnesses by showcasing interventions and resources relevant to each determinant. The concentration on representing the intersectionality of determinants provides additional support for multilevel interventions which consider the complex factors simulations shape mental health.

## Supplementary Information

**Additional file 1: Figure S1. **Excerpt from grey literature data extraction form.** Table S1. **Determinants of public mental health, by framework development stage.

## Data Availability

All data generated during this study are included in the published article and supplementary information. Notes from all consultations and workshops have been anonymised and cannot be attributed to individual participants. All data is stored on an encrypted, secured drive at UCL. Further information is available from the corresponding author.

## Abbreviations

NIHR: National Institute for Health and Care Research
PMH: Public mental health
SPHR: School for Public Health Research
