# Supplementary material for: Conceptualising public mental health: development of a conceptual framework for public mental health
Source: BMC Public Health. 2022 Jul 23;22:1407. doi: 10.1186/s12889-022-13775-9 (PMC9308351; doi:10.1186/s12889-022-13775-9)
Supplement: Supplementary file 1 — Additional file 1: Figure S1. Excerpt from grey literature data extraction form. Table S1. Determinants of public mental health, by framework development stage. [file 12889_2022_13775_MOESM1_ESM.docx]

**Table S1 Determinants of public mental health identified, by framework development stage**

**INDIVIDUAL**

| **STAGE 1** | | | **STAGE 2** | | **STAGE 3** |
| --- | --- | --- | --- | --- | --- |
| **STATE-OF-THE-ART REVIEW** | **GREY LITERATURE** | **PUBLIC MIND MAPS** | **POTENTIAL DETERMINANTS (FOR WORKSHOPS)** | **UPDATED DETERMINANTS (FOR SURVEY)** | **FINAL LIST OF DETERMINANTS** |
| DEMOGRAPHIC | | | | | |
| Economic inequality, individual-level poverty, and area deprivation | Income | Financial (in)security | Income (including financial (in)security) | Income (including financial insecurity, poverty, debt, food insecurity) | Income (including financial insecurity, poverty, debt, food insecurity) |
| Psychosocial burdens |  |  | Psychosocial burdens |  |  |
| Food insecurity |  |  | Food (in)security |  |  |
| Job insecurity, underemployment, unemployment | Employment (including un/underemployment, occupational position) | Employment (including having a job, fair conditions, job suitability and satisfaction, work-life balance) | Employment (un/under employment, occupational position, job conditions, satisfaction, work-life balance) | Employment (unemployment, occupational position, job conditions, satisfaction, work-life balance) | Employment (unemployment, occupational position, job conditions, satisfaction, work-life balance) |
|  | Work environment | Work environment |  |  |  |
| Poor housing quality and housing instability | Housing (including quality, stability, crowding) | Housing (including a safe place to call home) | Housing (including quality, stability, crowding, safe place to call home) | Housing (including quality, stability, crowding, a safe place to call home) | Housing (including quality, stability, crowding, a safe place to call home) |
| Low educational attainment | Education | Education (including accessibility, inclusion, quality, completion) | Education (including accessibility, inclusion, quality, completion, environment) | Education (including accessibility, inclusion, quality, completion, transitions, environment) | Education (including accessibility, inclusion, quality, completion, transitions, environment) |
|  | School environment | Educational environment |  |  |  |

| **STAGE 1** | | | **STAGE 2** | | **STAGE 3** |  |
| --- | --- | --- | --- | --- | --- | --- |
| **STATE-OF-THE-ART REVIEW** | **GREY LITERATURE** | **PUBLIC MIND MAPS** | **POTENTIAL DETERMINANTS (FOR WORKSHOPS)** | **UPDATED DETERMINANTS (FOR SURVEY)** | **FINAL LIST OF DETERMINANTS** | |
| LIFE EXPERIENCES AND OPPORTUNITIES | | | | | | |
|  | Life events and transitions (including bereavement, job loss, retirement, becoming a carer) | Life challenges and transitions (divorce, bereavement, job loss, retirement, etc.) | Life transitions (including bereavement, job loss, retirement, caring) | Life transitions (including bereavement, job loss, retirement, becoming a parent or carer) | Life transitions (including bereavement, job loss, retirement, becoming a parent or carer) | |
| Acculturation | Migration |  | Migration | Migration (including cultural differences, language barriers) | Migration (including cultural differences, language barriers) | |
|  |  | Hobbies and leisure | Hobbies and leisure time | Hobbies and leisure time | Hobbies and leisure time | |
| TRAUMA AND ADVERSITY | | | | | | |
| Adverse early life experiences: sexual, emotional, physical abuse | Trauma and adversity | Adverse childhood experiences | Trauma and adversity (including adverse childhood and adult experiences) | Trauma and adversity (including adverse childhood and adult experiences) | Adverse childhood experiences | |
| Adult life experiences: intimate partner violence, economic abuse |  | Abuse, adversity, and trauma |  |  | Adulthood trauma (including domestic violence) | |
| Lifetime traumas |  |  |  |  |  |  |
| History of rape or stalking |  |  |  |  |  |  |
| Physical assault |  |  |  |  |  |  |
|  | Bullying (including cyberbullying) | Bullying (including cyberbullying and fear) | Bullying (including cyberbullying) |  | Bullying | |

| **STAGE 1** | | | **STAGE 2** | | | **STAGE 3** |  |
| --- | --- | --- | --- | --- | --- | --- | --- |
| **STATE-OF-THE-ART REVIEW** | **GREY LITERATURE** | **PUBLIC MIND MAPS** | | **POTENTIAL DETERMINANTS (FOR WORKSHOPS)** | **UPDATED DETERMINANTS (FOR SURVEY)** | **FINAL LIST OF DETERMINANTS** | |
| PHYSICAL AND PSYCHOLOGICAL HEALTH | | | | | | | |
|  | Genetics and biological factors | Biological factors | | Genetics and biological factors | Genetics and biological factors | Genetics and biological factors | |
|  |  | Family history and genetics | |  |  |  |  |
| Stressors surrounding pre- and antenatal periods | Pre- and peri-natal factors |  | | Pre- and peri-natal factors | Prenatal and perinatal factors | Prenatal and perinatal factors | |
|  | Physical health (including illness, disability, frailty) | Physical health | | Physical health (including illness, disability, frailty) | Physical health (including illness, disability, frailty) | Physical health (including illness, disability, frailty) | |
| Stress | Health behaviours (including physical activity, healthy eating) | Nutrition | | Health behaviours (including physical activity, healthy eating) | Health behaviours (including physical activity, nutrition, sleep) | Health behaviours (including physical activity, nutrition, sleep) | |
|  |  | Self-care | | Self-care |  |  |  |
|  |  | Rest and relaxation | |  |  |  |  |
|  |  |  |  | Stress |  |  |  |
|  | Sleep | Sleep | | Sleep |  |  |  |

| **STAGE 1** | | | **STAGE 2** | | **STAGE 3** |
| --- | --- | --- | --- | --- | --- |
| **STATE-OF-THE-ART REVIEW** | **GREY LITERATURE** | **PUBLIC MIND MAPS** | **POTENTIAL DETERMINANTS (FOR WORKSHOPS)** | **UPDATED DETERMINANTS (FOR SURVEY)** | **FINAL LIST OF DETERMINANTS** |
| PERSONAL TRAITS | | | | | |
| Resilience | Resiliency | Coping resources and resilience and ability to deal with adversity | Resiliency (including coping resources, ability to deal with adversity, post traumatic growth) | Resilience (including coping resources, ability to deal with adversity, posttraumatic growth, ability to manage a full range of emotions, self-care) | Resilience (including coping resources, ability to deal with adversity, posttraumatic growth, ability to manage a full range of emotions, self-care) |
|  |  | Sense of worth and belonging | Sense of self-worth and belonging | Sense of self (including self-power and respected boundaries, sense of contribution and purpose, sense of self-worth and belonging, living by values) | Sense of self (including self-power and respected boundaries, sense of contribution and purpose, sense of self-worth and belonging, living by values) |
|  |  | Self-power and respected boundaries | Self-power and respected boundaries |  |  |
|  |  | Living by values | Living by values |  |  |
|  |  | Experiencing full spectrum of emotions | Experiencing full spectrum of emotions |  |  |
|  |  | Ability to self-regulate |  |  |  |
|  |  | Happiness and fulfilment | Happiness and fulfilment |  |  |
|  |  | Tolerance and compassion | Tolerance and compassion |  |  |
|  |  | Sense of contribution and purpose | Sense of contribution and purpose |  |  |
|  |  | Aspirations | Aspirations | Personal aspirations and ambitions | Personal aspirations and ambitions |
|  | Personality | Personality | Personality |  |  |
|  |  | Autonomy | Autonomy (including choice and control) | Individual autonomy (including choice, control and freedom) | Individual autonomy (including choice, control and freedom) |
|  |  | Choice and control |  |  |  |

| **STAGE 1** | | | **STAGE 2** | | **STAGE 3** |
| --- | --- | --- | --- | --- | --- |
| **STATE-OF-THE-ART REVIEW** | **GREY LITERATURE** | **PUBLIC MIND MAPS** | **POTENTIAL DETERMINANTS (FOR WORKSHOPS)** | **UPDATED DETERMINANTS (FOR SURVEY)** | **FINAL LIST OF DETERMINANTS** |
| IDENTITY | | | | | |
|  | Ethnicity | Ethnicity | Ethnicity and culture (including indigenous status) | Ethnicity and culture (including indigenous status) | Ethnicity and culture (including indigenous status) |
|  | Culture | Culture |  |  |  |
|  | Indigenous status |  |  |  |  |
|  | Gender and sex |  | Gender and sex | Gender, sex, gender identity, and sexual orientation | Gender, sex, gender identity, and sexual orientation |
|  | Gender identity and sexual orientation | Gender identity and sexual orientation | Gender identity and sexual orientation |  |  |
| Spirituality | Religiosity and spirituality |  | Religiosity and spirituality | Religion, spirituality, and faith | Religion, spirituality, and faith |
| Religious engagement |  |  |  |  |  |

**FAMILY**

| **STAGE 1** | | | **STAGE 2** | | **STAGE 3** |
| --- | --- | --- | --- | --- | --- |
| **STATE-OF-THE-ART REVIEW** | **GREY LITERATURE** | **PUBLIC MIND MAPS** | **POTENTIAL DETERMINANTS (FOR WORKSHOPS)** | **UPDATED DETERMINANTS (FOR SURVEY)** | **FINAL LIST OF DETERMINANTS** |
| **FAMILY STRUCTURE** | | | | | |
| Single motherhood status | Marital status |  | Marital and domestic partnership | Marriage, civil and domestic partnerships | Marriage, civil and domestic partnerships |
| Marital/domestic partnership status |  |  |  |  |  |
| Caregiving burden | Caring and care taking |  | Caring and care taking | Caring responsibilities | Caring responsibilities |
|  | Intergenerational advantage and disadvantage |  | Intergenerational advantage and disadvantage | Intergenerational advantage and disadvantage (including the economic and social circumstances we inherit from our parents) | Intergenerational (dis)advantage (including the economic and social circumstances we inherit from our parents) |
|  |  |  |  |  | Household composition (including changes like divorce or loss) |

| **STAGE 1** | | | **STAGE 2** | | **STAGE 3** |
| --- | --- | --- | --- | --- | --- |
| **STATE-OF-THE-ART REVIEW** | **GREY LITERATURE** | **PUBLIC MIND MAPS** | **POTENTIAL DETERMINANTS (FOR WORKSHOPS)** | **UPDATED DETERMINANTS (FOR SURVEY)** | **FINAL LIST OF DETERMINANTS** |
| FAMILY FUNCTIONING | | | | | |
|  | Parenting | Family and upbringing | Parenting | Early life attachment and parenting | Attachment |
|  |  |  | Early life attachment |  | Parenting |
|  | Early life attachment |  |  | Family relationships and connectivity | Family connectivity |
|  |  |  |  |  | Extended family relationships |
| Expectations of motherhood, wife, daughter |  |  |  |  | Discord and conflict (including different social, cultural, or generational values and expectations) |
| Expectations of father, husband, son |  |  |  |  |  |

**COMMUNITY**

| **STAGE 1** | | | **STAGE 2** | | **STAGE 3** |
| --- | --- | --- | --- | --- | --- |
| **STATE-OF-THE-ART REVIEW** | **GREY LITERATURE** | **PUBLIC MIND MAPS** | **POTENTIAL DETERMINANTS (FOR WORKSHOPS)** | **UPDATED DETERMINANTS (FOR SURVEY)** | **FINAL LIST OF DETERMINANTS** |
| SOCIAL ENVIRONMENT | | | | | |
| Social networks | Social networks | Social relationships and support network | Social network (social relationships, number of friends) | Social support and networks | Social support and networks |
| Bridging networks | Friendship (number, relationship quality) |  |  |  |  |
| Bonding networks |  |  |  |  |  |
| Social support | Social support |  | Social support (quality of relationships) |  |  |
| Social isolation | Loneliness | Loneliness | Loneliness |  |  |
| Social integration |  |  | Social engagement |  |  |
| Social engagement |  |  |  |  |  |
| Social exclusion | Social inclusion | Social inclusion and sense of community | Social inclusion and exclusion | Social inclusion and cohesion (including marginalisation, segregation, trust in community and institutions) | Social inclusion and cohesion (including marginalisation, segregation, trust in community and institutions) |
| Marginalization and segregation |  |  |  |  |  |
| Social inclusion |  |  |  |  |  |
| Social safety | Social fragmentation/social cohesion |  | Social fragmentation and cohesion (including marginalization and segregation) |  |  |
| Neighbourhood social capital |  |  | Neighbourhood social capital |  |  |
| Trust, safety |  | Safety | Trust |  |  |
| Civic engagement | Civic participation and engagement | Civic participation (voting, volunteering, charitable giving, etc.) | Civic participation (including voting, volunteering, charitable giving) | Civic engagement (including voting, volunteering, charitable giving) | Civic engagement (including voting, volunteering, charitable giving) |
|  |  | Cultural engagement |  |  |  |

| **STAGE 1** | | | **STAGE 2** | | **STAGE 3** |  |
| --- | --- | --- | --- | --- | --- | --- |
| **STATE-OF-THE-ART REVIEW** | **GREY LITERATURE** | **PUBLIC MIND MAPS** | **POTENTIAL DETERMINANTS (FOR WORKSHOPS)** | **UPDATED DETERMINANTS (FOR SURVEY)** | **FINAL LIST OF DETERMINANTS** |  |
| SOCIAL ENVIRONMENT (continued) | | | | | | |
| Information access |  | Mental health education and access to information | Mental health awareness | Mental health support in the workplace and educational settings | Mental health awareness |  |
|  |  | Mental health awareness |  | General practitioners' knowledge of mental health |  |  |
|  |  | Prevention | Prevention |  |  |  |
| SYSTEMS AND SERVICES (available, high-quality, accessible, connected) | | | | | | |
| Limited health care access |  | Health and social care (accessibility, responsiveness, appropriateness, quality) | Access to health and social care | Access to health and social care (including health promotion, mental illness prevention, crisis services) | Health and social care |  |
| Services |  |  |  |  |  |  |
| Health care provider availability |  |  |  |  |  |  |
| Neighbourhood crime and vandalism |  | Law enforcement (responsive, supportive, effective) | Criminal justice system | Criminal justice system (including mental health support for those in prison or leaving prison) | Criminal justice system (including mental health support for those in prison or leaving prison) |  |
|  |  | Crime and fear of |  |  |  |  |
|  |  | Violence and fear of |  |  |  |  |
|  |  | Access to amenities | Public and community services | Public and community services (including public transport, community spaces, legal support, creative outlets) | Public and community services (including public transport, community spaces, legal support, creative outlets) |  |
|  |  | Access to health-promoting activities (exercise, creative outlets, etc.) |  |  |  |  |

| **STAGE 1** | | | **STAGE 2** | | **STAGE 3** |  |
| --- | --- | --- | --- | --- | --- | --- |
| **STATE-OF-THE-ART REVIEW** | **GREY LITERATURE** | **PUBLIC MIND MAPS** | **POTENTIAL DETERMINANTS (FOR WORKSHOPS)** | **UPDATED DETERMINANTS (FOR SURVEY)** | **FINAL LIST OF DETERMINANTS** |  |
| GEOGRAPHIC and PHYSICAL ENVIRONMENT | | | | | | |
|  | Environmental factors (including air pollution, green space) | Environmental factors (including pollution) | Environmental factors (including air pollution, green space, clean water) | Environmental factors (including air and water quality, sanitation, noise, green space) | Built and natural environment (including air and water quality, sanitation, noise, green space, walkability, urban decay) |  |
|  |  | Green spaces |  |  |  |  |
| Built environment | Built environment | Built environment | Built environment | Built environment (including population density, walkability, urban decay) |  |  |
|  |  | Transport links |  |  |  |  |
|  | Population density and urbanicity (including remoteness) |  | Population density, urbanicity, and remoteness | Urban/rural/remote divides (including the "North-South" regional divide) | Urban/rural/remote differences |  |
|  | Ethnic density |  |  |  | North-South regional divide |  |
| Neighbourhood decay | Neighbourhood deprivation | Deprived neighbourhoods | Neighbourhood deprivation and decay | Neighbourhood deprivation and poverty | Neighbourhood deprivation |  |
| Neighbourhood affordability |  |  |  |  |  |  |
| Neighbourhood crime | Safety (including perceived safety) |  | Safety (including perceived safety, violence/crime and fear of, gang involvement, law enforcement) | Community safety (including perceived safety, violence/crime, fear, gang involvement, law enforcement) | Community safety (including perceived safety, violence/crime, fear, gang involvement, law enforcement) |  |
|  | Crime/violence (including gang involvement) |  |  |  |  |  |

**STRUCTURAL**

| **STAGE 1** | | | **STAGE 2** | | **STAGE 3** |
| --- | --- | --- | --- | --- | --- |
| **STATE-OF-THE-ART REVIEW** | **GREY LITERATURE** | **PUBLIC MIND MAPS** | **POTENTIAL DETERMINANTS (FOR WORKSHOPS)** | **UPDATED DETERMINANTS (FOR SURVEY)** | **FINAL LIST OF DETERMINANTS** |
| BROAD FACTORS | | | | | |
|  | Inequality | Equity | Inequality/ inequity | Inequality/ inequity | (In)equality and (in)equity |
|  |  | Social inequalities |  |  |  |
|  | Natural disasters | Climate change | Natural disasters | Natural disasters | Climate change |
|  | Climate change |  |  |  |  |
| War dislocation |  |  | Displacement (including forced migration due to conflict) | Displacement (including asylum seeking, refugee status, forced migration) | Displacement (including asylum seeking, refugee status, forced migration) |
| GOVERNMENT AND POLITICAL | | | | | |
| Global markets | Economic recession | Economy and economic inequalities | Economic recession | Economic recession | Economic conditions |
|  | Funding decisions |  | Taxation and funding decisions | Government policies and legislation (including funding decisions, taxation) | Government policies and legislation (including funding decisions, taxation) |
|  | Taxation |  |  |  |  |
| Policy | Policies and laws | Policies and laws | Policies and laws |  |  |
|  |  |  | Government agencies | The welfare system (including pensions, housing, disability, and unemployment benefits) | The welfare system (including pensions, housing, disability, and unemployment benefits) |
|  |  | Aid (fair benefits system, homeless shelters, food banks, advocacy, etc.) | Aid (fair benefits system, homeless shelters, food banks, advocacy etc.) |  |  |
| Political structures | Political climate | Political climate | Political structures and climate | Political structures and climate | Political structures and climate |
| Government agencies |  |  |  |  |  |
|  |  |  |  |  | Global politics and events |

| **STAGE 1** | | | **STAGE 2** | | **STAGE 3** |
| --- | --- | --- | --- | --- | --- |
| **STATE-OF-THE-ART REVIEW** | **GREY LITERATURE** | **PUBLIC MIND MAPS** | **POTENTIAL DETERMINANTS (FOR WORKSHOPS)** | **UPDATED DETERMINANTS (FOR SURVEY)** | **FINAL LIST OF DETERMINANTS** |
| NORMS and RIGHTS | | | | | |
| Discrimination and stigma | Discrimination and stigma | Discrimination and racism | Discrimination and stigma | Discrimination and stigma | Discrimination and stigma |
| Hypermasculinity | Social expectations and norms | Social expectations and norms | Social norms and expectations (including hypermasculinity, social roles) | Cultural and social norms (including societal expectations, public rhetoric, beliefs around mental health) | Cultural and social norms (including societal expectations, public rhetoric, beliefs around mental health) |
| Dominant culture |  |  | Dominant culture |  |  |
|  |  | Public rhetoric | Public rhetoric |  |  |
|  |  |  |  |  | Human rights and social justice |
| INDUSTRY | | | | | |
| Big business | Commercial determinants |  | Commercial determinants (including big business, capitalist structures) | Commercial determinants (including factors that influence health which stem from a profit motive, like big business and capitalist structures) | Commercial determinants (including factors that influence health which stem from a profit motive, like big business and capitalist structures) |
| Capitalist structures |  |  |  |  |  |
| Media and advertising | Media | Social media | Media and advertising (including social media and screen time) | Media and advertising (including social media, screen time) | Media and advertising (including social media, screen time) |
|  | Social media use | Media |  |  |  |
|  | Screen time |  |  |  |  |

**TOTAL DETERMINANTS IDENTIFIED AT EACH STAGE**

| **STATE-OF-THE-ART REVIEW** | **GREY LITERATURE** | **PUBLIC MIND MAPS** | **POTENTIAL DETERMINANTS (FOR WORKSHOPS)** | **UPDATED DETERMINANTS (FOR SURVEY)** | **FINAL LIST OF DETERMINANTS** |
| --- | --- | --- | --- | --- | --- |
| 56 determinants | 56 determinants | 67 determinants | 72 determinants | 48 determinants | 55 determinants |
